# Classification of circadian pain rhythms and pain characteristics in chronic pain patients: An observational study

**DOI:** 10.1097/MD.0000000000026500

**Published:** 2021-06-25

**Authors:** Yoichi Tanaka, Hayato Shigetoh, Gosuke Sato, Ren Fujii, Ryota Imai, Michihiro Osumi, Shu Morioka

**Affiliations:** a Graduate School of Health Sciences, Kio University; b Department of Rehabilitation, Nara Prefecture General Rehabilitation Center; c Neuro Rehabilitation Research Center, Kio University, Nara; d Department of Rehabilitation, Osaka Kawasaki Rehabilitation University, Osaka, Japan.

**Keywords:** chronic pain, circadian pain rhythms, neuropathic pain, pain management, psychological factors

## Abstract

This study aimed to perform cluster analysis in patients with chronic pain to extract groups with similar circadian rhythms and compare neuropathic pain and psychological factors among these groups to identify differences in pain-related outcomes. A total of 63 community-dwellers with pain lasting at least 3 months and Numerical Rating Scale scores of ≥2 were recruited from 3 medical institutions. Their pain circadian rhythms were evaluated over 7 days by measuring pain intensity at 6-time points per day using a 10-cm visual analog scale. Cluster analysis was performed using 6 variables with standardized visual analog scale values at 6-time points for individual participants to extract groups with similar pain circadian rhythms. The results of the Neuropathic Pain Symptom Inventory and psychological evaluations in each group were compared using the Kruskal–Wallis test. The results revealed 3 clusters with different circadian rhythms of pain. The total and evoked pain subscale Neuropathic Pain Symptom Inventory scores differed among the 3 clusters. The results suggest that a thorough understanding of circadian pain rhythms in chronic pain patients may facilitate the performance of activities of daily living and physical exercise from the perspective of pain management.

## 1 Introduction

The International Association for the Study of Pain defines chronic pain as “pain that extends beyond the expected period of healing or progressive pain due to non-cancer disease”.^[1]^ Persistent pain stimulation induces nervous system degeneration via various mechanisms, including abnormal excitation of the spinal dorsal horn^[2]^ and disruption of the pain control mechanism of the cerebral cortex.^[3]^ At present, approximately 20 million patients in Japan experience chronic pain,^[4]^ with 30% of them having a pain history of ≥5 years and still receiving treatment,^[5]^ resulting in soaring costs of medical care. In addition, chronic pain is closely associated with inactivity and reduced quality of life,^[6]^ which substantially hampers the affected individual's ability to live in the community.

Pain is known to be associated with psychological factors, such as catastrophic thinking, anxiety, and depression,^[7,8]^ and social factors, such as narrowing of social relationships and social roles.^[9,10]^ Furthermore, our previous study showed that pain is associated with an individual's social skills and social communication abilities.^[11]^ In patients experiencing chronic pain, psychosocial factors become intertwined with the pain pathology, complicating treatment. Therefore, at present, the treatment for chronic pain is aimed at improving patients’ quality of life and ability to perform activities of daily living.^[12]^ Additionally, instead of focusing on pain elimination, there is a focus on the importance of pain management, which includes understanding pain patterns while engaging in daily activities and physical exercise. In fact, McDonough et al reported that achieving life goals while managing pain led to improvements not only in pain intensity but also in self-efficacy and physical activity levels.^[13]^ Furthermore, community-dwelling chronic pain patients who perform more physical activity not only have lower pain levels but are also in a better psychological condition than those with lower activity levels.^[14]^ These findings highlight the importance of selecting appropriate approaches for daily activities and physical exercises while managing chronic pain in patients. However, to provide even more specific and efficient lifestyle guidance, the diurnal variations in the patient's chronic pain need to be studied.

The circadian rhythms of pain have received increasing attention in recent years. Studies have shown that different diseases exhibit different rhythms.^[15–19]^ One study of patients with diabetic and post-herpetic neuralgia revealed that pain was more intense in the evening and through the night than in the morning.^[20]^ In addition, we conducted a preliminary survey of the circadian rhythms of pain in a patient with brachial plexus injury, observing a U-shaped rhythm wherein pain intensity was higher when the patient woke up and went to bed but lower during the daytime.^[21]^ A thorough understanding of these circadian rhythms can facilitate the performance of daily activities and physical exercise based on the time of the day, leading to better pain management. Moreover, previous studies have observed different types of circadian rhythms, such as pain that increases in intensity over time or shows U-shaped patterns.^[17,20]^ Although other circadian patterns are presumed to exist, the factors associated with these patterns have not been identified. Furthermore, it is difficult to identify the factors that cause these patterns in studies of circadian rhythms by disease category because a wide variety of rhythms tend to get aggregated together.

Therefore, to identify factors associated with the circadian rhythms of pain, we focused not on the diseases but on the factors and mechanisms associated with pain, namely neuropathic pain (NP) and psychological factors. This is because in a study of patients with chronic low back pain who were classified into 2 groups based on pain mechanism, patients with NP exhibited more intense pain and more negative psychological states than those with nociceptive pain.^[22]^ Therefore, we thought that NP would also influence the circadian rhythms of pain. In addition, Wolf et al reported that in patients with fibromyalgia, the severity of loneliness in the morning affected the intensity of pain in the evening.^[23]^ This is because the psychological factor of loneliness may be contributing to the circadian rhythm more than the disease factor of fibromyalgia. Considering the findings of these studies, it seems possible that NP and psychological factors affect circadian rhythms, making it necessary to understand and classify the circadian rhythms of disease categories and of pain itself. Therefore, in the present study, we proposed 2 hypotheses: that there exist multiple patterns of pain circadian rhythms, and that NP and psychological factors are associated with these circadian rhythms. To test these hypotheses, we first performed cluster analysis on patients with chronic pain to extract groups with similar circadian rhythms, after which we compared NP and psychological factors among these groups to identify differences in these outcomes.

## 2 Methods

### 2.1 Ethics statement

The experimental protocol was approved by the Kio University Ethics Committee (approval number: H30-31), and the study protocol conformed to the Declaration of Helsinki (UMIN: 20141113-184337). Participants provided written consent to participate in the study after receiving an explanation of the procedures involved.

### 2.2 Participants

This study was carried out from April 2018 to December 2019. A total of 63 community-dwellers with pain lasting at least 3 months and Numerical Rating Scale (NRS) scores of ≥2 were recruited from 3 medical institutions (outpatient rehabilitation center, orthopedic clinic, and daycare facility). Specifically, we included patients who were able to travel indoors and outdoors with a level of functioning of more than modified independence and who were not receiving regular treatment at medical institutions. We excluded patients with a diagnosis of dementia and mental illness. We determined our sample size with reference to those of previous studies that performed cluster analysis; they had sample sizes of approximately 60.^[24,25]^

### 2.3 Procedure

The participants’ demographic data (sex, age, disease, major pain sites, duration of pain, medication status, and employment), pain characteristics, and psychological status were assessed using questionnaires that the respondents were administered in a single session. Subsequently, their pain circadian rhythms were evaluated.

### 2.4 Pain circadian rhythm

Pain circadian rhythm was evaluated over 7 days. Pain intensity was measured at 6-time points per day (wake-up, 9:00, 12:00, 15:00, 18:00, and 21:00) using a 10-cm visual analog scale (VAS) drawn on paper. The evaluation was performed by the participants themselves, and participants who could not perform the evaluations at the exact time points because of their work or lifestyle were asked to record their measurements within a 1-hour window of the time point (e.g., if they found it difficult to perform the evaluation at 15:00, they were asked to perform it between 14:00 and 16:00). For the analysis, we used a 7-day average for each of the 6-time points. Participants showing VAS score variations of <1 in the 6-time points on all 7 days were considered to not show circadian pain rhythms and excluded from the study (n = 3).

### 2.5 Measures

The Neuropathic Pain Diagnostic Questionnaire (DN4) was used to determine NP, the Short-Form McGill Pain Questionnaire 2 (SFMPQ2) was used as a multidimensional assessment of pain intensity, the Neuropathic Pain Symptom Inventory (NPSI) was used to assess the severity of NP, and the Michigan Body Map (MBM) was used to evaluate the pain site. Psychological states were assessed using the Hospital Anxiety and Depressions Scale (HADS) for depression and anxiety, the Pain Catastrophizing Scale (PCS-4) for pain catastrophizing thoughts, and the UCLA Loneliness Scale (Japanese version) for loneliness.

(1) Neuropathic Pain Diagnostic Questionnaire
  The DN4 is an evaluation scale developed to classify pain into NP and non-NP.^[26]^ It consists of 10 questions related to NP (e.g., “Do you have a burning pain?” and “Do you have pain like an electric shock?). If there are positive responses to 4 or more items, the pain is judged as NP.
(2) Short-Form McGill Pain Questionnaire 2
  The SFMPQ2 consists of 22 questions that are categorized into 4 sub-items: continuous pain, intermittent pain, affective descriptors, and NP.^[27]^ Each item was answered on an NRS of 11 points, and the higher the score, the more severe the pain. SFMPQ2 shows good internal consistency (SFMPQ2-total: Cronbach's α = 0.86).^[27]^ There were significant correlations between SFMPQ2-total and other functional assessments (VAS: *ρ* = 0.54, SFMPQ2-total: *ρ* = 0.79).^[27]^
(3) Neuropathic Pain Symptom Inventory
  The NPSI is a 10-item questionnaire related to NP (e.g., “Do you have burning spontaneous pain?” and “Do you have attacks of pain, like a knife stabbing?”). These 10 items can be classified into the following sub-items: spontaneous pain, attacks of pain, provoked pain, and abnormal sensations.^[28]^ These symptoms were rated by the patients on an 11-point NRS by selecting the number that best described their average pain within the last 24 hours (range, 0–10, where 10 indicates maximum pain). The higher the total score, the more severe the symptoms of NP.
(4) Michigan Body Map
  The MBM is a self-administered rating scale in which the patient marks the area of current pain from 35 boxes representing body regions shown on an image of the body.^[29]^ The greater the number of marks, the greater the number of painful areas.
(5) Hospital Anxiety and Depressions Scale
  The HADS contains 14 items and 2 subscales. The 2 subscales independently assess depression and anxiety.^[28]^ Higher scores indicate more severe anxiety and depression. HADS-anxiety showed good internal consistency (HADS-anxiety: Cronbach's *α* = 0.80), while HADS-depression did not show good internal consistency (HADS-depression: Cronbach's *α* = 0.50–0.61).^[30]^
(6) Pain Catastrophizing Scale
  The PCS-4 is a shorter version of the 13-item PCS and contains 4 items. Higher scores indicate more severe catastrophic thinking. PCS-4 showed good internal consistency (Cronbach's *α* = 0.86).^[29]^ There were significant correlations between PCS-4 and PCS-13 (*r* = 0.96).^[31]^
(7) The Japanese Revised UCLA Loneliness Scale
  This scale was translated and revised by Moroi,^[32]^ tested in participants across a wide age range (teenagers to those aged over 65 years), and then named the Japanese Revised UCLA Loneliness Scale. The scale includes items, such as “You stand alone from other people” and “No one knows you well.” It consists of 20 items, including 10 reversed items. The items are rated on a scale from 1 to 4 (1 = “absolutely disagree” to 4 = “very strongly agree”).

### 2.6 Data analyses

The demographic variables of all subjects were presented as absolute numbers and percentages (sex, age, major pain site, pain onset period, analgesic use, and employment status). Additionally, the chi-square test was used to compare the demographic characteristics of the patients classified according to the medical institution from which they were recruited. Correlations between pain intensity (SFMPQ2) and pain-related factors (NPSI, each psychological factor) were examined in all participants using Spearman's rank correlation coefficients. Further, cluster analysis (mixed distribution model) was performed using 6 variables with standardized (Z-score) VAS values (7-day mean) at 6-time points for individual participants in order to extract groups with similar pain rhythms. The number of clusters, determined using the Bayesian Information Criterion (BIC) value as a reference, was the number between 2 and 7 with the smallest BIC value.^[33]^ Subsequently, the period of evaluation of the participants’ circadian rhythm of pain and the medical institutions from which they were recruited were compared among the clusters using the chi-square test. Subsequently, age, sex, NP rate, major pain site, analgesic use, MBM, employment status, and disease rate in each group were compared using the Kruskal–Wallis test and chi-squared test. The NP ratio was calculated from the DN4 results. Finally, the results of the SFMPQ2, NPSI, and psychological evaluation in each group were compared using the Kruskal–Wallis test, and post-hoc assessments were performed using the Steel–Dwass test. These statistical analyses were performed using HAD (ver. 14.101)^[34]^ with a significance level of 5%.

## 3 Results

### 3.1 Demographic data

A total of 63 participants were recruited for this study, and statistical analysis was performed on 56 of them (7 participants were excluded either because of missing data [n = 4] or because they did not show circadian pain rhythms [n = 3]). Table 1 shows the demographic characteristics of the participants. The major pain sites, including the upper limb and lower limb, accounted for <80% of the total pain area. With respect to the chronicity of pain, 35.7% of the participants had pain for more than 3 months but less than 1 year, 28.6% had pain for more than 1 year but less than 5 years, and 35.7% had pain for more than 5 years. Less than 50% of the participants used analgesics, and 47.5% were employed. Additionally, there were no significant differences between the participants’ demographic characteristics when they were classified according to the medical institutions from which they were recruited.

**Table 1:** Characteristics of the participants (n = 56).

Characteristics, n (%)	
Gender (male)	24 (42.9)
Age (yr), mean ± SD	63.3 ± 18.1
Main pain area	
Neck	1 (1.8)
Low back	13 (23.2)
Upper limb	16 (28.6)
Lower limb	26 (46.4)
Pain duration	
3 mo–1 yr	20 (35.7)
1–5 yr	16 (28.6)
over 5 yr	20 (35.7)
Analgesic use	27 (48.2)
Working	21 (47.5)
Outcome measures, mean (SD)	
Neuropathic Pain Diagnostic Questionnaire, n (%)	20 (35.7)
Short-Form McGill Pain Questionnaire 2-total	37.2 ± 33.3
Neuropathic Pain Symptom Inventory-total	16.4 ± 15.0
Michigan Body Map	5.9 ± 5.2
Hospital Anxiety and Depressions Scale-total	13.6 ± 6.9
Pain Catastrophizing Scale-4	10.4 ± 3.0
The Japanese Revised UCLA Loneliness Scale (Loneliness)	36.3 ± 10.5

SD = standard deviation.

### 3.2 Correlation analysis

Table 2 shows the results of the correlation analysis of the scores of the outcomes in all participants. Significantly strong correlations were observed between SFMPQ2 and NPSI (rs = 0.76, *P* < .001). In addition, the psychological evaluations PCS-4 and HADS were positively correlated with SFMPQ2 (PCS-4: rs = 0.29, *P* < .05; HADS: rs = 0.39, *P* < .01).

**Table 2:** Correlations between pain related outcomes and SF-MPQ2.

	NPSI	PCS-4	HADS	Loneliness
SF-MPQ2	0.76^∗∗∗^	0.29^∗^	0.39^∗∗^	0.12

HADS = Hospital Anxiety and Depressions Scale-total values, Loneliness, The Japanese Revised UCLA Loneliness Scale-total values, NPSI = Neuropathic Pain Symptom Inventory-total values, PCS-4 = Pain Catastrophizing Scale-total values, SF-MPQ2 = Short-Form McGill Pain Questionnaire 2-total values.

∗ *P* < .05.

∗∗ *P* < .01.

∗∗∗ *P* < .001.

### 3.3 Cluster analysis

**Figure 1:**
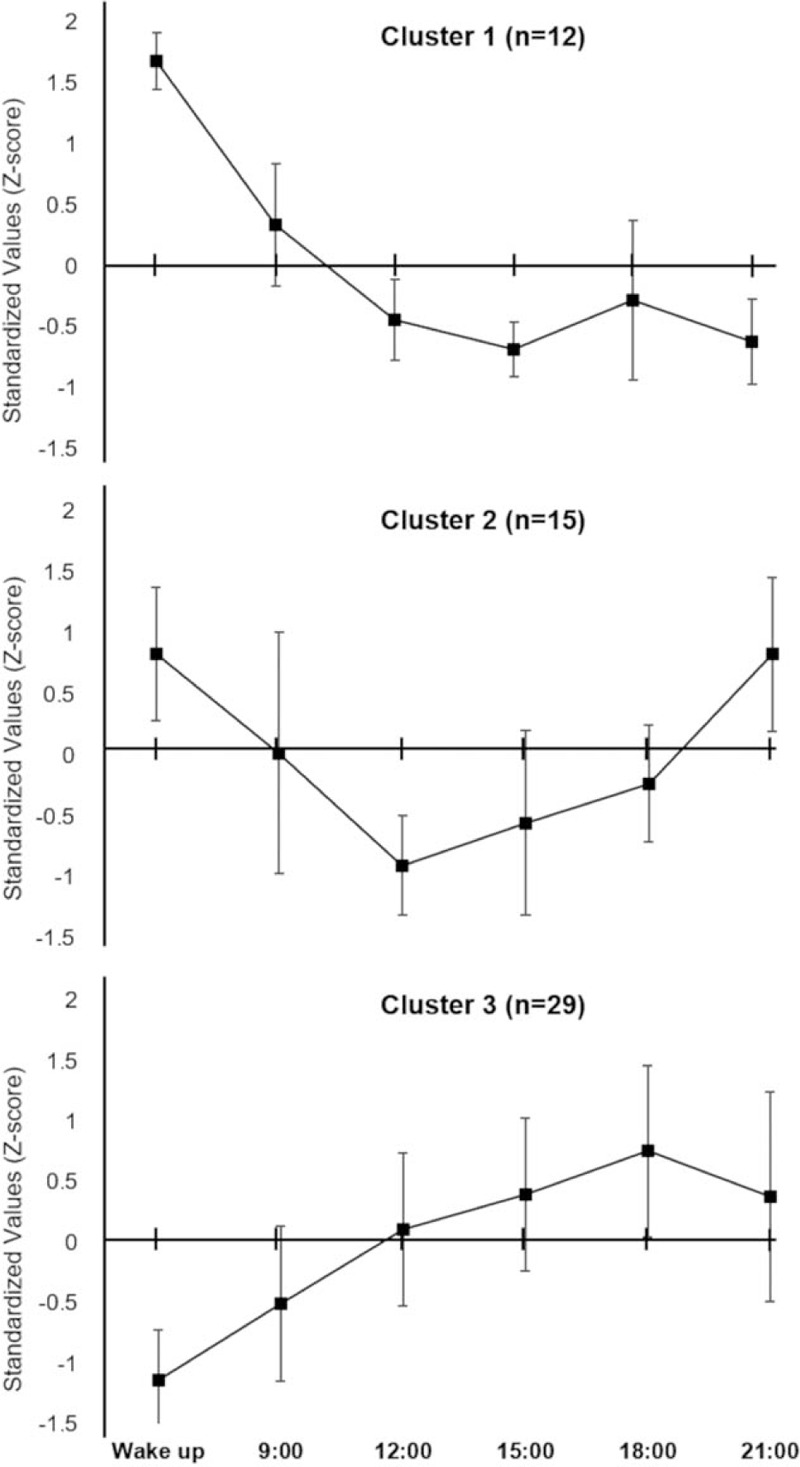
Rhythm-type classification of circadian pain rhythms. Three clusters with different characteristics were extracted through cluster analysis. CL1 showed the highest VAS score at waking, but the VAS score tended to decrease with time. In CL2, the VAS score was high at waking and decreased during the day, but gradually increased after 15:00. CL3 showed a trend of gradual increase of VAS score over time. Each data point is displayed with an error bar. CL = cluster, VAS = visual analog scale.

Table 3 shows the BIC values in 2–7 clusters. Cluster analysis yielded 3 similar clusters of circadian pain rhythms (Fig. 1). Cluster 1 (CL1) showed the highest VAS score at waking, but the VAS score tended to decrease with time. In cluster 2 (CL2), the VAS score was high at waking and decreased during the day as in CL1, but gradually increased after 15:00 and tended to be the same as that at waking by 21:00. Cluster 3 (CL3) showed a trend of gradual increase of VAS score over time, contrary to CL1. Tables 4 and 5 show the characteristics of each cluster and the results of the intergroup comparisons. There were no significant differences in all factors among the 3 groups. The percentage of NP was 24.1% in CL3, which was lower than that in the other 2 groups (CL1: 41.7%, CL2: 53.3%), but there were no significant differences among the 3 groups. There was no significant difference in disease rates among the groups.

**Table 3:** BIC value in each cluster number.

	2CL	3CL	4CL	5CL	6CL	7CL
BIC	811.1	804.7	806.4	808.7	821.7	848.7

BIC = Bayesian Information Criterion, CL = cluster.

**Table 4:** Comparison between clusters based on evaluation period and medical institution.

Evaluation period and medical institution, n (%)				
	CL1 (n = 12)	CL2 (n = 15)	CL3 (n = 29)	*P* value
Evaluation period				*P* = .163
April–June	3 (25)	5 (33.3)	10 (34.5)	
July–September	1 (8.3)	3 (20)	9 (31)	
October–December	1 (8.3)	4 (26.7)	5 (17.2)	
January–March	7 (58.3)	3 (20)	5 (17.2)	
Medical institutions				*P* = .383
Outpatient rehabilitation center	6 (50)	9 (60)	10 (34.5)	
Orthopedic clinic	5 (41.7)	5 (33.3)	12 (41.4)	
Daycare facility	1 (8.3)	1 (6.7)	7 (24.1)	

CL = cluster.

**Table 5:** Characteristics and disease ratio of each group.

Characteristics and disease ratio, n (%)				
	CL1 (n = 12)	CL2 (n = 15)	CL3 (n = 29)	*P* value
Gender (male)	7 (58.3)	7 (46.7)	10 (34.5)	*P* = .562
Age (yr), mean ± SD	69.7 ± 12.2	59.0 ± 18.3	62.8 ± 19.8	*P* = .371
Neuropathic pain ratio	5 (41.7)	8 (53.3)	7 (24.1)	*P* = .276
Main pain area				*P* = .088
Neck	0	0	1 (3.4)	
Low back	2 (16.7)	4 (13.3)	7 (24.1)	
Upper limb	4 (33.3)	9 (60)	3 (10.3)	
Lower limb	6 (50)	2 (13.3)	18 (62.1)	
Analgesic use	8 (66.7)	7 (46.7)	12 (41.4)	*P* = .521
Michigan Body Map, mean ± SD	5.9 ± 4.4	5.3 ± 6.7	6.2 ± 4.9	*P* = .400
Working	3 (25)	6 (40)	12 (41.4)	*P* = .812
Disease ratio				*P* = .932
Spinal cord disease	5 (41.7)	5 (33.3)	10 (34.5)	
Locomotor disorders	6 (50)	4 (26.7)	11 (37.9)	
Lower back pain	1 (8.3)	1 (6.7)	5 (17.2)	
Stroke	0	2 (13.3)	0	
Cause unknown	0	3 (20)	3 (10.3)	

Spinal cord disease includes spinal cord injury, spinal canal stenosis, cervical spondylosis, etc. Locomotor disorders includes those due to osteoarthritis, after artificial joint replacement, fracture etc. CL = cluster, SD = standard deviation.

### 3.4 Comparisons among groups

**Figure 2:**
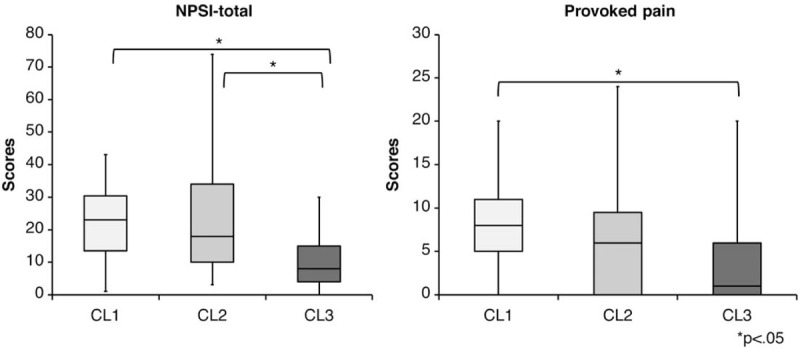
The results of multiple comparisons. CL1 and CL2 showed higher total NPSI scores than CL3. For provoked pain, CL1 showed higher scores than CL3. CL = cluster, NPSI = Neuropathic Pain Symptom Inventory.

The intergroup comparisons showed significant differences in the total NPSI score and the sub-item score for provoked pain (Table 6). After the post-hoc test, CL1 and CL2 showed significantly higher total NPSI scores than CL3 (CL1 vs CL2, *P* = .595; CL1 vs CL3, *P* = .020; CL2 vs CL3, *P* = .036; 95% CI: CL1 = 13.6–30.8, CL2 = 11.5–41.2, CL3 = 6.9–15.1). For provoked pain, CL1 showed significantly higher scores than CL3 (CL1 vs CL2, *P* = .302; CL1 vs CL3, *P* = .044; CL2 vs CL3, *P* = .305; 95% CI: CL1 = 4.3–11.9, CL2 = 1.5–12.5, CL3 = 0.6–6.5) (Fig. 2).

**Table 6:** Between-groups comparison of pain-related outcomes.

Pain-related outcomes, mean ± SD				
	CL1 (n = 12)	CL2 (n = 15)	CL3 (n = 29)	*P* value
SF-MPQ2-total	32.5 ± 14.7	55.4 ± 48.0	32.5 ± 31.8	*P* = .255
Continuous pain	15.2 ± 8.3	18.8 ± 14.6	13.5 ± 11.5	*P* = .451
Intermittent pain	6.8 ± 6.5	15.1 ± 13.6	8.2 ± 12.3	*P* = .233
Affective descriptors	2.5 ± 3.0	7.7 ± 11.0	5.1 ± 5.3	*P* = .412
Neuropathic pain	8.0 ± 5.1	13.9 ± 13.1	7.5 ± 8.5	*P* = .215
NPSI-total	22.2 ± 12.8	23.9 ± 21.1	10.3 ± 8.4	*P* = .007
Spontaneous pain	5.8 ± 4.6	4.9 ± 7.4	3.2 ± 3.7	*P* = .126
Attacks of pain	4.5 ± 5.9	5.7 ± 5.9	2.0 ± 2.7	*P* = .094
Provoked pain	8.1 ± 5.6	7.0 ± 7.7	3.5 ± 5.0	*P* = .033
Abnormal sensations	3.8 ± 3.4	6.3 ± 6.6	2.2 ± 2.6	*P* = .099
HADS	11.6 ± 4.9	14.6 ± 9.0	13.8 ± 6.4	*P* = .958
PCS-4	9.8 ± 2.5	10.2 ± 4.2	10.7 ± 2.6	*P* = .553
Loneliness	36.8 ± 10.5	40.0 ± 12.1	34.1 ± 9.3	*P* = .334

HADS = Hospital Anxiety and Depressions Scale-total values, Loneliness = The Japanese Revised UCLA Loneliness Scale, NPSI = Neuropathic Pain Symptom Inventory-total values, PCS-4 = Pain Catastrophizing Scale, SF-MPQ2 = Short-Form McGill Pain Questionnaire 2-total values.

## 4 Discussion

The present study investigated the relationship between the types of circadian pain rhythms and the factors associated with these rhythms among community-dwelling chronic pain patients. The most common site of pain was the lower extremity (46%), and the total number of patients experiencing lower-extremity and upper-extremity pain accounted for <80% of the participants, with 35.7% of the participants experiencing NP. Analgesics were used by 48% of the participants, and 47% were employed. The cluster analysis showed 3 clusters of circadian pain rhythms with different characteristics.

In this study, we investigated the circadian rhythms of pain that had persisted for 3 months or longer regardless of disease. Most previous studies on circadian pain rhythms have examined only certain diseases, but even patients with the same disease may show a wide variety of pain types, indicating the difficulty in understanding these rhythms by disease category. Our results showed that the disease distributions were not significantly different among the 3 clusters. However, the clusters showed different circadian patterns with unique characteristics. Conventionally, pain has been evaluated and classified based on its mechanism.^[35]^ Our results also indicate that it is appropriate to evaluate circadian rhythms across disease categories since these rhythms present in patterns that are difficult to explain based on disease characteristics. Thus, in chronic pain patients, circadian rhythms need to be evaluated individually. Understanding individual circadian rhythms may help in developing treatment strategies, such as adjusting daily activities and physical activities. Further, although we did not control analgesic use, previous studies demonstrated that even though analgesics reduced pain during the day, they did not change the circadian pain rhythm that was present before analgesic administration.^[20]^ Consistent with these findings, our results did not show any significant differences in analgesic use among the 3 clusters, indicating that analgesics have minimal impact on circadian pain rhythms.

Considering psychological factors, although our correlation analysis showed significant positive correlations between SFMPQ2 and PCS-4 and HADS, there were no differences among the clusters. Previous studies have found that psychological factors both increase and decrease pain intensity,^[36–38]^ and the results of our correlation analysis suggest that psychological factors are likely to modify pain intensity at each time period. However, the existence of circadian rhythms of the psychological factors themselves has also been suggested,^[39]^ and psychological states may fluctuate both within and between days. Therefore, assessment of psychological factors at a 1-time point, as was done in the present study, may not demonstrate their correlations with circadian pain rhythms. In future studies, it will be necessary to investigate the circadian rhythms of psychological factors as well and to examine their interrelationships with the circadian pain rhythms.

Notably, the NPSI total and provoked pain subscale scores exhibited significant differences among clusters. The NPSI total scores of CL1 and CL2 were significantly higher than that of CL3. CL1 and CL2 exhibited higher proportions of NP than CL3, although the differences were not significant (CL1: 41%, CL2: 53%, CL3: 24%), and while CL3 showed the lowest pain intensity at wake-up, CL1 and CL2 both showed high pain intensities at this time. Thus, NP and its severity are associated with circadian rhythms of pain intensity at wake-up. Since nervous and neuroendocrine system regulations have been suggested to contribute to circadian pain rhythms,^[20]^ it is possible that the NPSI score, which can assess the severity of NP, is associated with these rhythms. Moreover, previous studies have found that patients with NP show lower melatonin secretion at night than healthy subjects. This may cause sleep disorders, which may promote pain sensitivity.^[40]^ While we did not measure melatonin secretion or sleep disorders, we surmise that the results of high pain intensities at wake-up in CL1 and CL2 in this study are also related to these factors.

The NPSI provoked pain subscale score was significantly higher in CL1 than in CL3. A study on spinal cord injuries classified patients into 5 clusters of different sensory symptoms based on NPSI score distributions.^[41]^ Thus, NP presents with a wide variety of sensory symptoms, and it is possible that the symptom of provoked pain itself is associated with the circadian pain rhythm. Further, while CL1 and CL2 exhibited similarly high pain intensities at wake-up, pain intensity increased from afternoon to night in CL2 but remained low in CL1. Unlike spontaneous pain and attacks of pain, patients with provoked pain can avoid the stimulus that causes the pain; thus, the circadian pattern for CL1 could have been affected by the means of pain avoidance, creating a different pattern from CL2.

On the other hand, the circadian rhythm for CL3 showed a gradual increase in pain intensity from wake-up to nighttime. CL3 showed the lowest proportion of NP (24%), which may indicate a strong role of the nociceptive element. This is thought to indicate that a gradual increase in physical activity throughout the day exacerbates the pain. While physical activity has been reported to alleviate pain,^[42–44]^ intense physical activity has also been found to exacerbate pain.^[45]^ Because overactivity may exacerbate pain and modulate the circadian rhythm when thinking about the management of chronic pain, it is important to consider the interrelationship between the circadian rhythms of pain and physical activity.

This study had several limitations. First, concerning the classification of circadian rhythms, previous studies have reported that the intensity of chronic pain fluctuates between days.^[46]^ However, the classifications in the present study were based on mean VAS scores at each time point over 7 days, making it impossible to examine variations between days. Second, it has been suggested that exogenous factors, such as temperature, atmospheric pressure, and humidity, affect circadian pain rhythms.^[20]^ Because the subjects of the present study were not examined over the same period, we cannot rule out the impact of exogenous factors on circadian pain rhythms. Third, we did not evaluate factors, such as neuroendocrine secretions, sleep, and physical activity levels; thus, we can only speculate how these relate to the circadian rhythms of pain. Tasks for future studies include evaluation of the neuroendocrine system, sleep, and physical activity alongside circadian pain rhythms and further clarification of the factors that are associated with circadian pain rhythms.

In conclusion, we investigated the circadian pain rhythms in community-living chronic pain patients and were able to extract 3 clusters of different circadian pain rhythms through cluster analysis. In addition, we found differences in the total NPSI score and the subscale score for evoked pain in the 3 clusters. Rather than understanding circadian pain rhythms from disease classification, rhythms should be assessed individually, considering the nature of the pain, such as NPSI. Furthermore, this study's results suggest that a thorough understanding of circadian pain rhythms in chronic pain patients may facilitate the introduction of treatment strategies, such as activities of daily living and physical exercise.

## Acknowledgments

We thank all the participants from the 3 medical institutions.

## Author contributions

**Conceptualization:** Yoichi Tanaka, Shu Morioka.

**Data curation:** Yoichi Tanaka, Hayato Shigetoh, Gosuke Sato, Ren Fujii, Ryota Imai.

**Formal analysis:** Yoichi Tanaka.

**Funding acquisition:** Yoichi Tanaka.

**Investigation:** Yoichi Tanaka.

**Methodology:** Yoichi Tanaka.

**Supervision:** Ryota Imai, Michihiro Osumi, Shu Morioka.

**Writing – original draft:** Yoichi Tanaka.

## References

[1] Merskey H Bogduk N. Taxonomy Classification of Chronic Pain. 2nd ed 1994;Seattle: IASP Press, 209–214.

[2] Lefaucheur JP Antal A Ahdab R. The use of repetitive transcranial magnetic stimulation (rTMS) and transcranial direct current stimulation (tDCS) to relieve pain. Brain Stimul 2008;1:337–44. 20633392 10.1016/j.brs.2008.07.003

[3] Naro A Milardi D Russo M. Non-invasive brain stimulation, a tool to revert maladaptive plasticity in neuropathic pain. Front Hum Neurosci 2016;10:376. 27512368 10.3389/fnhum.2016.00376 PMC4961691

[4] Yabuki S Ushida T Takeshita K. A national survey of patients with chronic pain in Japan [in Japanese]. Clin Orthop (Rinsho Seikeigeka) 2012;47:127–34.

[5] Nakamura M Toyama Y Nishiwaki Y Ushida T. Prevalence and characteristics of chronic musculoskeletal pain in Japan: a second survey of people with or without chronic pain. J Orthop Sci 2014;19:339–50. 24504984 10.1007/s00776-013-0525-8 PMC3960485

[6] Leadley RM Armstrong N Reid KJ Allen A Misso KV Kleijnen J. Healthy aging in relation to chronic pain and quality of life in Europe. Pain Pract 2014;14:547–58. 24138082 10.1111/papr.12125

[7] Edwards RR Kronfli T Haythornthwaite JA Smith MT McGuire L Page GG. Association of catastrophizing with interleukin-6 responses to acute pain. Pain 2008;140:135–44. 18778895 10.1016/j.pain.2008.07.024 PMC2659503

[8] Meier ML Stämpfli P Humphreys BK Vrana A Seifritz E Schweinhardt P. The impact of pain-related fear on neural pathways of pain modulation in chronic low back pain. Pain Rep 2017;2:e601. 29392216 10.1097/PR9.0000000000000601 PMC5741307

[9] Farin E. The reciprocal effect of pain catastrophizing and satisfaction with participation in the multidisciplinary treatment of patients with chronic back pain. Health Qual Life Outcomes 2015;13:163. 26420426 10.1186/s12955-015-0359-5 PMC4588313

[10] Macdonald G Leary MR. Why does social exclusion hurt? The relationship between social and physical pain. Psychol Bull 2005;131:202–23. 15740417 10.1037/0033-2909.131.2.202

[11] Tanaka Y Nishi Y Nishi Y Osumi M Morioka S. Uncovering the influence of social skills and psychosociological factors on pain sensitivity using structural equation modeling. J Pain Res 2017;10:2223–31. 28979161 10.2147/JPR.S143342 PMC5602447

[12] Wickson-Griffiths A Kaasalainen S Herr K. Interdisciplinary approaches to managing pain in older adults. Clin Geriatr Med 2016;32:693–704. 27741964 10.1016/j.cger.2016.06.013

[13] McDonough SM Tully MA Boyd A. Pedometer-driven walking for chronic low back pain: a feasibility randomized controlled trial. Clin J Pain 2013;29:972–81. 23446066 10.1097/AJP.0b013e31827f9d81 PMC4895187

[14] Tawashy AE Eng JJ Lin KH Tang PF Hung C. Physical activity is related to lower levels of pain, fatigue and depression in individuals with spinal-cord injury: a correlational study. Spinal Cord 2009;47:301–6. 18936771 10.1038/sc.2008.120 PMC3095632

[15] Allen KD Coffman CJ Golightly YM Stechuchak KM Keefe FJ. Daily pain variations among patients with hand, hip, and knee osteoarthritis. Osteoarthr Cartilage 2009;17:1275–82. 10.1016/j.joca.2009.03.021 19410670

[16] Bellamy N Sothern RB Campbell J. Rhythmic variations in pain perception in osteoarthritis of the knee. J Rheumatol 1990;17:364–72. 2332859

[17] Bellamy N Sothern RB Campbell J Buchanan WW. Rhythmic variations in pain, stiffness, and manual dexterity in hand osteoarthritis. Ann Rheum Dis 2002;61:1075–80. 12429538 10.1136/ard.61.12.1075 PMC1753960

[18] Hegarty RS Treharne GJ Stebbings S Conner TS. Fatigue and mood among people with arthritis: carry-over across the day. Health Psychol 2016;35:492–9. 26867041 10.1037/hea0000321

[19] Stone AA Broderick JE Porter LS Kaell AT. The experience of rheumatoid arthritis pain and fatigue: examining momentary reports and correlates over one week. Arthritis Care Res 1997;10:185–93. 9335630 10.1002/art.1790100306

[20] Gilron I Bailey JM Vandenkerkhof EG. Chronobiological characteristics of neuropathic pain: clinical predictors of diurnal pain rhythmicity. Clin J Pain 2013;29:755–9. 23370066 10.1097/AJP.0b013e318275f287

[21] Tanaka Y Osumi M Sato G. Relief of daily chronic pain through physical activity: a case study of right brachial plexus damage-related pain. Occup Ther 2019;38:117–22.

[22] Spahr N Hodkinson D Jolly K Williams S Howard M Thacker M. Distinguishing between nociceptive and neuropathic components in chronic low back pain using behavioural evaluation and sensory examination. Musculoskelet Sci Pract 2017;27:40–8. 28637600 10.1016/j.msksp.2016.12.006 PMC5329124

[23] Wolf LD Davis MC Yeung EW. The within-day relation between lonely episodes and subsequent clinical pain in individuals with fibromyalgia: mediating role of pain cognitions. J Psychosom Res 2015;79:202–6. 25637526 10.1016/j.jpsychores.2014.12.018 PMC4496321

[24] Knoerl R Chornoby Z Smith EML. Estimating the frequency, severity, and clustering of SPADE symptoms in chronic painful chemotherapy-induced peripheral neuropathy. Pain Manag Nurs 2018;19:354–65. 29503217 10.1016/j.pmn.2018.01.001

[25] Manca M Ferraresi G Cosma M Cavazzuti L Morelli M Benedetti MG. Gait patterns in hemiplegic patients with equinus foot deformity. Biomed Res Int 2014;2014:939316. 24967417 10.1155/2014/939316 PMC4016931

[26] Bouhassira D Attal N Alchaar H. Comparison of pain syndromes associated with nervous or somatic lesions and development of a new Neuropathic Pain Diagnostic Questionnaire (DN4). Pain 2005;114:29–36. 15733628 10.1016/j.pain.2004.12.010

[27] Maruo T Nakae A Maeda L. Translation and reliability and validity of a Japanese version of the revised Short-Form McGill Pain Questionnaire (SF-MPQ-2). Pain Res 2013;28:43–53.

[28] Bouhassira D Attal N Fermanian J. Development and validation of the Neuropathic Pain Symptom Inventory. Pain 2004;108:248–57. 15030944 10.1016/j.pain.2003.12.024

[29] Hassett C Brummett A Clauw K. The Michigan Body Map and its use in assessing the American College of Rheumatology survey criteria for fibromyalgia. Arthritis Rheum 2011;63: Suppl 10: 939.21128258

[30] Hatta H Higashi A Yashiro H. A validation of the hospital anxiety and depression scale. Jpn J Psychosom Med 1998;38:309–15.

[31] Bot AGJ Becker SJE Bruijnzeel H. Creation of the abbreviated measures of the pain catastrophizing scale and the short health anxiety inventory: the PCS-4 and SHAI-5. J Musculoskelet Pain 2014;22:145–51.

[32] Moroi H. Dimensions of the revised UCLA Loneliness Scale [in Japanese]. Jinbun Ronsyu 1991;42:23–51.

[33] Schwarz G. Estimating the dimension of a model. Ann Statist 1978;6:461–4.

[34] Shimizu H. An introduction to the statistical free software HAD: suggestions to improve teaching, learning and practice data analysis [in Japanese]. J Media Inf Commun 2016;1:59–73.

[35] Woolf CJ Bennett GJ Doherty M. Towards a mechanism-based classification of pain? Pain 1998;77:227–9. 9808347 10.1016/S0304-3959(98)00099-2

[36] Domenech J Sanchis-Alfonso V López L Espejo B. Influence of kinesiophobia and catastrophizing on pain and disability in anterior knee pain patients. Knee Surg Sports Traumatol Arthrosc 2013;21:1562–8. 23081711 10.1007/s00167-012-2238-5

[37] Jaremka LM Fagundes CP Glaser R Bennett JM Malarkey WB Kiecolt-Glaser JK. Loneliness predicts pain, depression, and fatigue: understanding the role of immune dysregulation. Psychoneuroendocrinology 2013;38:1310–7. 23273678 10.1016/j.psyneuen.2012.11.016 PMC3633610

[38] Shuchang H Mingwei H Hongxiao J. Emotional and neurobehavioural status in chronic pain patients. Pain Res Manag 2011;16:41–3. 21369540 10.1155/2011/825636 PMC3052406

[39] Blume C Garbazza C Spitschan M. Effects of light on human circadian rhythms, sleep and mood. Somnologie (Berl) 2019;23:147–56. 31534436 10.1007/s11818-019-00215-x PMC6751071

[40] Fatima G Sharma VP Verma NS. Circadian variations in melatonin and cortisol in patients with cervical spinal cord injury. Spinal Cord 2016;54:364–7. 26572605 10.1038/sc.2015.176

[41] Soler MD Moriña D Rodríguez N. Sensory symptom profiles of patients with neuropathic pain after spinal cord injury. Clin J Pain 2017;33:827–34. 27977425 10.1097/AJP.0000000000000467

[42] Parker RS Lewis GN Rice DA McNair PJ. Is motor cortical excitability altered in people with chronic pain? A systematic review and meta-analysis. Brain Stimul 2016;9:488–500. 27133804 10.1016/j.brs.2016.03.020

[43] Di Santo AD Asci F Suppa A. Pain-motor integration and chronic pain: one step ahead. Clin Neurophysiol 2018;129:1051–2. 29530491 10.1016/j.clinph.2018.02.005

[44] Sato G Osumi M Morioka S. Effects of wheelchair propulsion on neuropathic pain and resting electroencephalography after spinal cord injury. J Rehabil Med 2017;49:136–43. 28101562 10.2340/16501977-2185

[45] Naugle KM Ohlman T Naugle KE Riley ZA Keith NR. Physical activity behavior predicts endogenous pain modulation in older adults. Pain 2017;158:383–90. 28187102 10.1097/j.pain.0000000000000769

[46] Bartley EJ Robinson ME Staud R. Pain and fatigue variability patterns distinguish subgroups of fibromyalgia patients. J Pain 2018;19:372–81. 29253551 10.1016/j.jpain.2017.11.014 PMC5869098

